# Anti-PLA2R antibody reduction as a predictor of treatment response in primary membranous nephropathy: a retrospective study

**DOI:** 10.1590/2175-8239-jbn-2025-0228en

**Published:** 2025-12-19

**Authors:** Khuram Bashir, Muhammad Rashid Asghar, Nayyar Saleem, Muhammad Shahzil, Aimon Riaz, Muhammad Nauman Hashmi, Ahmed Sheraz

**Affiliations:** 1 Multan Institute of Kidney Diseases, Multan, Punjab, Pakistan.; 2 Ministry of National Guard Health Affairs, Hemodialysis Care Project, Jeddah, Saudi Arabia.; 3 Punjab Employees Social Security Institution, Narowal, Punjab, Pakistan.

**Keywords:** Glomerulonephritis, Membranous, Phospholipase A2 Receptor antibody, Glomerular Diseases, Prognosis, Remission, Spontaneous

## Abstract

**Introduction:**

Primary membranous nephropathy (PMN) is a leading cause of nephrotic syndrome in adults. Although proteinuria has been the conventional guiding parameter in disease monitoring, its limitations as a non-specific and delayed marker have led to the appeal of anti-phospholipase A2 receptor antibodies (PLA2R) as specific and sensitive indicators of disease activity and response to treatment.

**Methods:**

This retrospective cohort study included 60 patients with biopsy-proven PMN and positive baseline anti-PLA2R antibodies who underwent treatment at the Multan Institute of Kidney Diseases. Anti-PLA2R levels were measured at baseline and at the 6-month follow-up using enzyme-linked immunosorbent assay (ELISA). Clinical remission was assessed using the standard proteinuria and renal function criteria. Statistical analyses included receiver operating characteristic (ROC) curves, logistic regression, and correlation assessments.

**Results:**

Baseline anti-PLA2R levels were significantly lower in patients who achieved remission (p = 0.047), and 6-month levels showed a strong correlation with treatment response (p < 0.001). ROC analysis demonstrated good predictive accuracy with area under curve (AUC) of 0.707 (baseline) and 0.815 (6-month), identifying optimal remission cutoffs at 125.5 RU/mL and 37.75 RU/mL, respectively. Logistic regression analysis confirmed the baseline albumin level, 6-month anti-PLA2R, and proteinuria reduction as independent predictors of remission.

**Conclusion:**

Anti-PLA2R antibody levels at baseline and 6 months serve as robust predictors of treatment response in PMN and outperform proteinuria in early remission assessment. These findings support the integration of anti-PLA2R monitoring into standard clinical practice for personalized management and therapy adjustment in PMN.

## Introduction

Primary membranous nephropathy (PMN) is a leading cause of nephrotic syndrome in adults, and is characterized by immune complex deposition in the glomerular basement membrane, resulting in proteinuria and potential progression to end-stage renal disease (ESRD)^1^. Accurate prognostic markers are crucial for guiding clinical management. However, the reliance on proteinuria alone has significant limitations. While proteinuria is a hallmark of PMN, it is a nonspecific marker influenced by extrinsic factors, such as dietary protein intake, hemodynamic fluctuations, and concurrent illnesses, reducing its reliability for disease monitoring^2^. The need for more precise biomarkers has led to growing interest in anti-phospholipase A2 receptor (PLA2R) antibodies, which have significantly advanced the diagnostic and prognostic assessment of PMN.

The identification of PLA2R as a major target antigen in PMN has improved the landscape of diagnostic nephrology. Anti-PLA2R antibodies are detected in approximately 85% of PMN cases, making them highly specific biomarkers for the disease^3^. Unlike proteinuria, which reflects downstream glomerular damage, anti-PLA2R antibodies are directly involved in the pathogenesis of PMN, offering a more accurate representation of disease activity. Their levels correlate with clinical course, including seropositivity during active disease, declining titers in remission, and reappearance during relapse^4^. This dynamic relationship makes anti-PLA2R antibodies ideal biomarkers for disease monitoring and risk stratification.

Proteinuria can remain elevated even after successful treatment, leading to prolonged immunosuppression. This is because they may not accurately correlate with the underlying immune activity that drives disease. For instance, studies have shown that proteinuria can persist despite the resolution of immune complex deposition^5^. Conversely, anti-PLA2R antibodies provide a more accurate reflection of disease activity, with studies showing that a 50% reduction in antibody levels within two months of treatment initiation is associated with favorable outcomes^6^. Furthermore, higher baseline levels of anti-PLA2R antibodies have been linked to a poorer prognosis and faster progression to ESRD, highlighting their utility in risk stratification and personalized treatment planning^7,8^.

PMN is treated with immunosuppressive therapies; however, the lack of reliable biomarkers to monitor treatment efficacy complicates clinical decision-making. The measurement of anti-PLA2R antibodies offers a promising solution, as it provides early insights into treatment response. For example, a 90% reduction in anti-PLA2R levels may justify immunosuppressive therapy withdrawal, thereby minimizing the risks associated with prolonged immunosuppression^9^.

Despite these advancements, a critical gap remains in our understanding of the correlation between anti-PLA2R antibody levels and treatment outcomes, particularly in diverse populations. Most studies have been conducted in Western cohorts, leaving the influence of genetic, environmental, and lifestyle factors in other regions largely unexplored. Metaanalyses support the diagnostic and prognostic value of anti-PLA2R antibodies, demonstrating their high specificity and sensitivity in differentiating PLA2R-related PMN from non-PLA2R cases and in predicting spontaneous remission^10,11,12^.

This study aimed to address these gaps by investigating the relationship between anti-PLA2R antibody levels and clinical outcomes in patients with PMN in the Multan Region of Pakistan. We hypothesized that anti-PLA2R antibody titers correlate with disease activity, treatment response, and long-term renal outcomes, thereby providing a reliable tool for personalized disease monitoring. Additionally, we aimed to compare treatment efficacy across different immunosuppressive regimens in relation to antibody levels, generating region-specific insights into the management of PMN. By bridging these gaps, this study sought to refine the prognostic and therapeutic frameworks for PMN in diverse populations. This study used a retrospective cohort design in accordance with the STROBE guidelines.

## Methods

### Inclusion and Exclusion Criteria

This retrospective cohort study was conducted at the Multan Institute of Kidney Diseases. The inclusion criteria were: diagnosis based on renal biopsy and immunostaining findings consistent with PMN; positive serum anti-PLA2R antibody at baseline; diagnosed and treated between August 2017 and June 2023 at Multan Institute of Kidney Diseases (MIKD); patients on immunosuppressive therapy, particularly those classified as moderate or high risk for disease progression (i.e., unlikely to undergo spontaneous remission); availability of anti-PLA2R antibody titers at baseline and at the 6-month follow-up; and willingness to participate and comply with study protocol requirements.

The exclusion criteria for the research were: positive for infections known to cause secondary MN (hepatitis B or C positive); any patient with a diagnosed active malignancy during evaluation or follow-up; exposure to known toxins or medications associated with secondary MN (e.g., NSAIDs, gold salts, and penicillamine); missing either baseline or 6-month follow-up anti-PLA2R antibody levels; and patients unwilling or unable to comply with follow-up or study protocol.

Selection bias was minimized by including all eligible patients who were consecutively diagnosed with biopsy-proven PMN and positive PLA2R between August 2017 and June 2023. Measurement bias was reduced using standard enzyme-linked immunosorbent assay (ELISA) protocols and laboratory personnel who were blinded to clinical outcomes.

### Patients and Blood Samples

**Figure 1.**
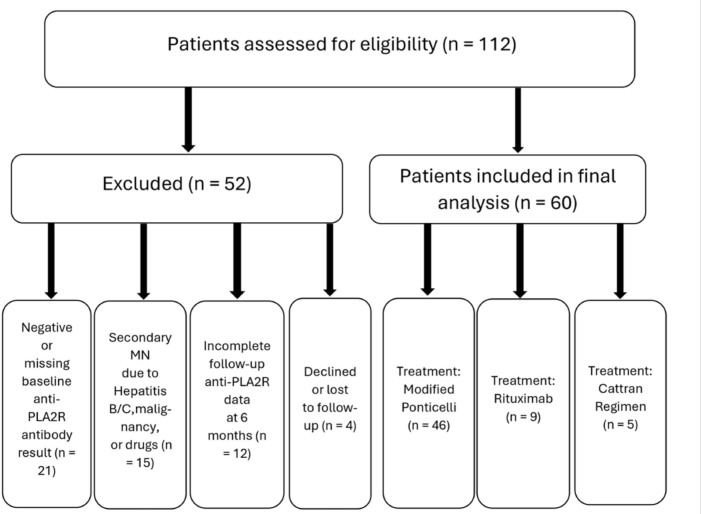
Of the 112 patients assessed for eligibility, 52 were excluded due to negative or missing anti-PLA2R antibody results, secondary causes of membranous nephropathy, incomplete data, or loss to follow-up. The final cohort included 60 patients, all of whom met the inclusion criteria and were analyzed. Patients were stratified into three treatment groups: Modified Ponticelli (n = 46), Rituximab (n = 9), and Cattran (n = 5).

Of the 112 patients assessed for eligibility, 52 were excluded due to negative or missing anti-PLA2R antibody results, secondary causes of membranous nephropathy, incomplete follow-up, or loss to follow-up. A total of 60 patients met the inclusion criteria and were analyzed. These were further stratified by treatment regimen into modified Ponticelli (n = 46), Rituximab (n = 9), and Cattran (n = 5) groups. The entire patient selection and treatment stratification process is outlined in Figure 1. This study was reviewed and approved as IRB-exempt by the Institutional Review Board of Indus Hospital and Health Network (IHHN), Pakistan, under exemption category 4 for secondary research involving existing clinical data and specimens without direct subject contact. The research adhered to the ethical principles outlined in the Declaration of Helsinki and the Belmont Report, and all data were handled in accordance with established standards of responsible conduct of research.

### Treatment Regimes

Treatment regimens were decided in collaboration with consultant nephrologists after considering what the patients wanted. Written informed consent was obtained from all patients. A modified Ponticelli regimen (MPR) was administered to 46 patients. Nine patients were administered rituximab and five were administered the Cattran regime.

### Remission Criteria

Complete remission (CR) was considered when the patient showed 24-h urine proteinuria of 0.3 g/d or less with stable or improved renal function from baseline on follow-up evaluations, while partial remission (PR) was considered if there was 24-h urine proteinuria of <3.5 g/d with a reduction of at least 50% alongside a stable renal function. No response was considered if there was 24-h urine proteinuria of >3.5g/d or worsening serum creatinine^13^.

### Quantitative Detection of PLA2R Antibodies

Serum samples were analyzed using quantitative ELISA.

### Statistical Analysis

Statistical analysis included descriptive statistics to summarize the baseline and follow-up variables. Chi-square tests were used to evaluate the relationships between binary variables whereas independent t-tests were used to check for mean differences between the remission and non-remission groups for continuous variables. Receiver operating characteristic (ROC) curve analysis evaluated the anti-PLA2R level predictive performance, and Youden’s index was used to determine the optimal cut-off values. Logistic regression was used to identify independent risk factors for remission status, and Pearson’s correlation coefficient was used to evaluate the magnitude and pattern of clinical variable relationships with remission outcomes. For categorical analysis, baseline anti-PLA2R titers were classified as low (<125.5 RU/mL) or high (≥125.5 RU/mL). The sample size was modest, especially in the non-MPR treatment group, which limited the statistical power of subgroup analysis.

## Results

### Patient Characteristics

The baseline characteristics of the 60 patients included in this study are summarized in Tables 1 and 2. The cohort consisted of 75.0% males (n = 45). Most patients were classified as having stage 1 CKD (68.3%, n = 41).

#### Categorical Variables

**Table 1:** Description of categorical variables

Variable	Category	Frequency (n)	Percent (%)
Gender	Male	45	75.0
	Female	15	25.0
CKD Stage	Stage 1	41	68.3
	Stage 2	13	21.7
	Stage 3	5	8.3
	Stage 4	1	1.7
IFTA	N/A	24	40.0
	Mild	31	51.7
	Moderate	5	8.3
Treatment Regimen	Modified Ponticelli regimen (MPR)	46	76.7
	Rituximab	9	15.0
	Cattran	5	8.3
Hypertension	No	45	75.0
	Yes	15	25.0
Baseline Anti-PLA2R Categories	Low Positive	31	51.7
	High Positive	29	48.3

#### Continuous variables

**Table 2:** Description of continuous variables

Parameter	Mean	Standard deviation
Age (years)	33.13	12.15
Baseline eGFR (mL/min/1.73m^2^)	107.32	41.71
Baseline Anti-PLA2R (RU/mL)	200.84	182.02
Baseline Proteinuria (g/day)	8.17	4.07
Baseline Albumin (g/dL)	2.17	0.66
Baseline Creatinine (mg/dL)	0.96	0.64
6-Month eGFR (mL/min/1.73m^2^)	105.12	39.38
6-Month Creatinine (mg/dL)	1.02	0.86
6-Month Anti-PLA2R (RU/mL)	48.87	80.64
6-Month Proteinuria (g/day)	4.43	3.89
6-Month Albumin (g/dL)	3.37	0.90

Interstitial fibrosis and tubular atrophy (IFTA) were mild in 51.7% (n = 31) of the patients and moderate in 8.3% (n = 5). Hypertension was present in 25.0% (n = 15) of the patients. No missing data were observed for the primary variables used in this analysis.

### Analysis Between Different Treatment Groups

In terms of treatment, 76.7% (n = 46) of the patients received MPR, 15.0% (n = 9) received rituximab, and 8.3% (n = 5) were treated with Cattran. Baseline anti-PLA2R antibody levels were categorized as low positive in 51.7% (n = 31) and high positive in 48.3% (n = 29) of patients.

The mean baseline eGFR (EPI-CKD) was 107.32 ± 41.71 mL/min, while mean baseline proteinuria was 8.17 ± 4.07 g/day. The mean serum albumin level at baseline was 2.17 ± 0.66 g/dL, and the mean baseline creatinine level was 0.96 ± 0.64 mg/dL. The mean baseline anti-PLA2R level was 200.84 ± 182.02 RU/mL.

#### Six-Month follow-up biomarkers

At six-month follow-up, the mean eGFR was 105.12 ± 39.38 mL/min, and the mean proteinuria level decreased to 4.43 ± 3.89 g/day. The mean serum albumin increased to 3.37 ± 0.90 g/dL, while the mean anti-PLA2R level decreased to 48.87 ± 80.64 RU/mL.

### FACTORS FOR REMISSION

#### Bivariate Analysis

Chi-square tests were used to assess categorical variables. No significant associations were found between remission and treatment type (χ^2^ = 1.85, p = 0.397), sex (χ^2^ = 1.09, p = 0.296), IFTA (χ^2^ = 1.09, p = 0.579), or hypertension (χ^2^ = 0.02, p = 0.881). However, baseline anti-PLAR categories showed a statistically significant association with remission (χ^2^ = 9.67, p = 0.002).

#### Comparison of continuous variables

Independent t-tests were used to compare continuous variables between remission and non-remission groups. Baseline anti-PLA2R levels were significantly lower in the remission group (t = –2.03, p = 0.047), whereas baseline albumin levels were significantly higher (t = 2.24, p = 0.029). However, age (t = 1.15, p = 0.253), CKD stage (t = –0.92, p = 0.363), baseline eGFR (t = 0.18, p = 0.86), baseline proteinuria (t = –0.37, p = 0.714), and baseline creatinine level (t = –0.24, p = 0.811) were not significantly different between groups (Table 3).

**Table 3:** Significance testing and correlation analysis of clinical variables with remission in primary membranous nephropathy

Variable	Test statistic	p-value	Correlation coefficient	p-value
Treatment	1.85	0.397	–0.081	0.539
Gender	1.09	0.296	0.135	0.305
IFTA	1.09	0.579	0.079	0.550
Hypertension	0.02	0.881	–0.019	0.884
Age in years	1.15	0.253	0.138	0.294
CKD Stage	–0.92	0.363	–0.098	0.458
eGFR (EPI-CKD)	0.18	0.860	–0.013	0.924
Baseline Anti-PLA2R (RU/mL)	–2.03	0.047	–0.359	0.005
Baseline Proteinuria (g/day)	–0.37	0.714	–0.024	0.855
Baseline Albumin (g/dL)	2.24	0.029	0.265	0.040
Baseline Creatinine (mg/dL)	–0.24	0.811	–0.070	0.597
GFR at 6 months (mL/min)	0.82	0.418	0.101	0.442
Creatinine at 6 months (mg/dL)	–1.36	0.180	–0.186	0.155
Anti-PLA2R at 6 months (RU/mL)	–3.67	0.001	–0.547	<0.001
Proteinuria at 6 months (g/day)	–8.45	<0.001	–0.866	<0.001
Albumin at 6 months (g/dL)	6.56	<0.001	0.663	<0.001

#### Six-month follow-up analysis

At six months, significant differences were observed in the follow-up anti-PLA2R levels (t = –3.67, p = 0.001), proteinuria (t = –8.45, p < 0.001), and albumin levels (t = 6.56, p < 0.001), all of which were strongly associated with remission. Changes in proteinuria from baseline to six months were also significantly associated with remission (t = 4.37, p < 0.001). However, the changes in anti-PLA2R (t = –0.58, p = 0.564) and GFR (t = –0.8, p = 0.429) were not statistically significant.

### Correlation Analysis

Pearson’s correlation coefficients were calculated to assess the relationship between remission and clinical variables. Baseline anti-PLA2R levels were negatively correlated with remission (r = –0.359, p = 0.005), whereas baseline albumin levels showed a positive correlation (r = 0.265, p = 0.040). At six months, strong correlations were observed between remission and follow-up anti-PLA2R levels (r = –0.547, p < 0.001), proteinuria (r = –0.866, p < 0.001), and albumin (r = 0.663, p < 0.001). Additionally, changes in proteinuria from baseline to six months demonstrated a significant positive correlation with remission (r = 0.484, p < 0.001).

Other variables, including age, sex, CKD stage, eGFR, IFTA, hypertension, complications, and treatment, did not show statistically significant correlations with remission (p > 0.05).

The significance and correlation findings are given in detail in Table 3.

### ROC Curve Analysis

#### Baseline anti-PLA2R antibody levels

**Figure 2.**
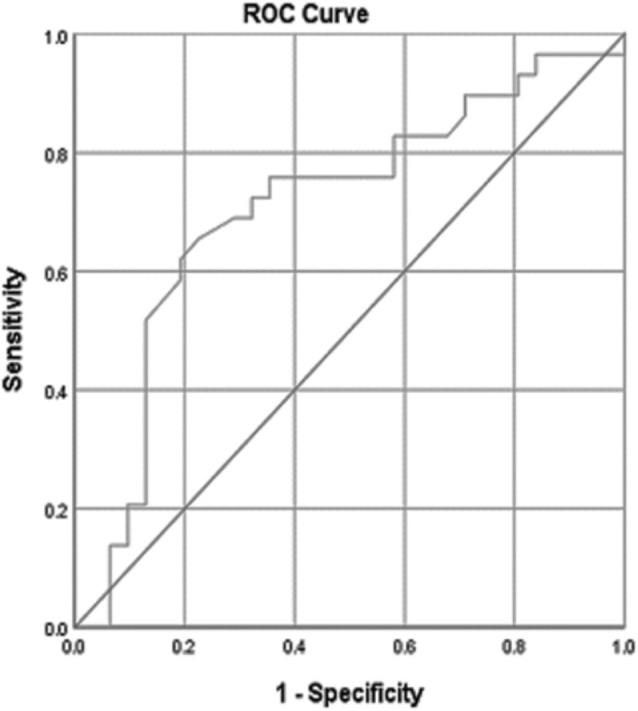
ROC curve showing baseline Anti-PLA2R levels as a predictor of remission in idiopathic membranous nephropathy. The AUC was 0.707 (95% CI: 0.570–0.845, p = 0.006), indicating moderate diagnostic accuracy. An optimal cutoff of 125.5 RU/mL provided 69.0% sensitivity and 71.0% specificity.

The ROC curve was used to evaluate baseline anti-PLA2R antibody levels for predicting remission in patients with idiopathic membranous nephropathy (IMN). The AUC was 0.707 (95% CI: 0.570–0.845), indicating moderate discriminatory power and significant predictive ability (p = 0.006), as shown in Figure 2. Using Youden’s index, the optimal cutoff for remission prediction was 125.5 RU/mL, which yielded sensitivity and specificity of 69.0% and 71.0%, respectively.

#### 6-month follow-up anti-PLA2R antibody levels

**Figure 3.**
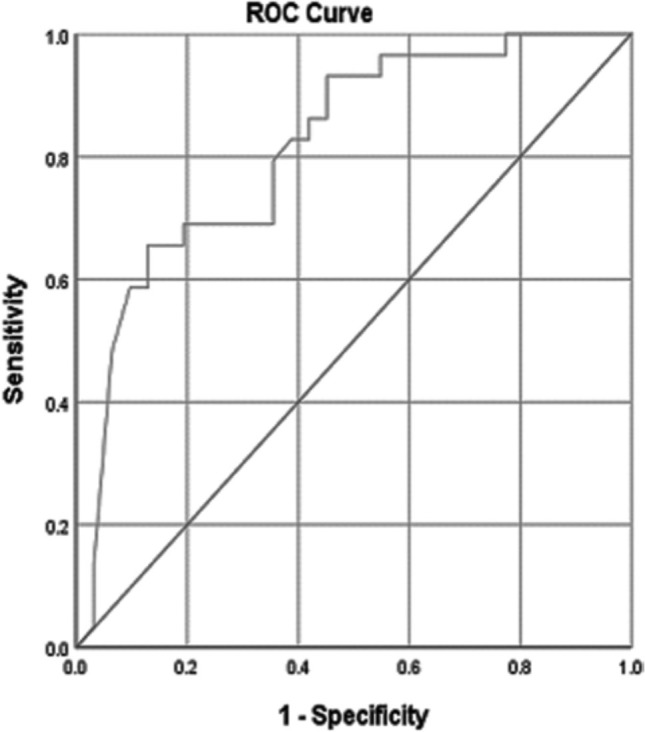
ROC curve evaluating 6-month anti-PLA2R levels as a predictor of remission in idiopathic membranous nephropathy. AUC = 0.815 (95% CI: 0.706–0.923, p < 0.001), indicating strong diagnostic accuracy. The optimal cutoff of 37.75 RU/mL provided 93.1% sensitivity and 54.8% specificity.

ROC analysis for 6-month anti-PLA2R levels showed an AUC of 0.815 (95% CI: 0.706–0.923), indicating strong diagnostic accuracy (p < 0.001), as shown in Figure 3. Using Youden’s index, the optimal cut-off was determined to be 37.75 RU/mL, which provided a sensitivity of 93.1% and specificity of 54.8%.

### Regression Analysis

Multivariate logistic regression was used to identify key predictors of remission in patients with IMN. A higher baseline serum albumin level was associated with a 167% increase in remission odds per g/dL (p = 0.041, Exp(B) = 2.669), which increased 12-fold at six months (p < 0.001). Lower 6-month anti-PLA2R levels also increased the chances of remission, with each 1 RU/mL reduction increasing the odds by 2.7% (p = 0.009, Exp(B) = 0.973). Conversely, higher baseline Log anti-PLA2R levels reduced remission odds by 83.5% (p = 0.012, Exp(B) = 0.165), indicating its value as a biomarker. Additionally, each 1 g/day reduction in proteinuria improved remission odds by 32% (p = 0.001, Exp(B) = 1.319). The results are presented in Table 4.

**Table 4:** Results from the regression analysis of significant variables

Predictor variable	p-value	Exp(B)
Baseline Albumin (g/dL)	0.041	2.669
6-Month Anti-PLA2R (RU/mL)	0.009	0.973
Baseline Anti-PLA2R	0.012	0.165
6-Month Albumin (g/dL)	<0.001	11.990
Change in Proteinuria (g/day)	0.001	1.319

## Discussion

This study underscores the pivotal role of anti-PLA2R antibody levels in predicting treatment response and long-term outcomes in patients with PMN in Multan, Pakistan. Our findings support global evidence highlighting the utility of anti-PLA2R antibodies as biomarkers for disease activity and remission, while offering region-specific insights into treatment efficacy and prognostic stratification^11,14,15^.

### Anti-PLA2R Antibodies as a Prognostic Biomarker

We found that baseline anti-PLA2R antibody levels were significantly lower in patients who achieved remission (p = 0.047), corroborating previous studies indicating that higher baseline titers were associated with poorer outcomes^5,16^. ROC curve analysis confirmed the predictive power of anti-PLA2R levels, with a baseline AUC of 0.707 (p = 0.006) and an optimal cutoff of 125.5 RU/mL (sensitivity 69.0%, specifically 71.0%). Notably, the 6-month follow-up levels had an even stronger discriminatory value (AUC = 0.815, p < 0.001), with a cutoff of 37.75 RU/mL (sensitivity, 93.1%; specificity, 54.8%). These results are consistent with those of Huang et al.^17^, who reported an AUC of 0.970 for PLA2R-IgG4 for predicting remission, thus reinforcing the relevance of antibody monitoring in diverse populations.

### Correlation with Clinical Parameters

The significant mean reductions in anti-PLA2R levels (–152.02 ± 165.91 RU/mL) and proteinuria (–3.74 ± 5.40 g/day) over six months reflect the close relationship between antibody titers and disease activity. Logistic regression identified baseline serum albumin levels (odds ratio (OR) = 2.669, p = 0.041) and 6-month anti-PLA2R levels (OR = 0.973, p = 0.009) as independent predictors of remission. These results echo those of Dahan et al.^18^, who found that serological remission often precedes clinical response, supporting early treatment adjustment based on antibody levels. Interestingly, the change in anti-PLA2R levels alone was not a significant predictor, suggesting that absolute thresholds were more clinically reliable than relative changes.

### Treatment Efficacy Across Regimens

In our cohort, 76.7% received the modified Ponticelli regimen (MPR), 15.0% rituximab, and 8.3% the Cattran regimen. Although no statistically significant difference in remission rates was observed across the treatment groups (p = 0.397), the consistent reduction in anti-PLA2R levels and proteinuria across all groups indicated a broad immunosuppressive efficacy. These findings are in line with those of Huang et al.^17^, who reported remission rates similar to those of rituximab and cyclophosphamide (74% vs. 67.5%, p = 0.114). Similarly, the RI-CYCLO randomized trial demonstrated that both therapies achieved comparable remission rates at 24 months, with cyclophosphamide showing a higher rate of complete remission at 12 months^19,20,21,22^. However, the small sample size of non-MPR regimens limits definitive conclusions and underscores the need for larger comparative studies in South Asian populations.

### ROC Curve Analysis and Clinical Utility

ROC analysis yielded moderate-to-high predictive values for anti-PLA2R levels, with AUCs of 0.707 (baseline) and 0.815 (6-month). This means that at baseline, there was a 70.7% chance and at six months an 81.5% higher chance that patients achieving remission would have lower anti-PLA2R levels than those without remission, underscoring the biomarker’s predictive value. Cutoff values of 125.5 RU/mL at baseline and 37.75 RU/mL at follow-up offered optimal sensitivity and specificity trade-offs. These results mirror the findings from Western cohorts and confirm that anti-PLA2R dynamics remain clinically relevant across populations. Our study fills a critical gap in the literature by contributing with data from a South Asian setting where such evidence has been sparse.

Furthermore, this study supports the growing consensus that proteinuria, although long used as a monitoring tool, has limitations owing to its delayed response and non-specific nature^19^. In contrast, anti-PLA2R antibodies provide realtime insights into disease activity, enabling earlier and more precise therapeutic decisions, including escalation, de-escalation, and discontinuation of immunosuppression.

### Recent Advances and Comparative Insights

Advances in immunopathology have substantially deepened our understanding of PMN, steering clinical practice towards precision medicine paradigms guided by biomarkers, such as anti-PLA2R antibodies^23^. Recent studies have emphasized the integration of serological markers with genetic predispositions, particularly PLA2R1 genetic polymorphisms, suggesting that combined serological and genetic profiling could significantly refine risk stratification^24,25^. Polymorphisms within the PLA2R1 gene have been associated with variations in antibody production and disease severity, implying that patients’ genetic backgrounds might influence therapeutic outcomes independently of serological titers^8^. Incorporating such genetic information into clinical algorithms could enhance the prediction accuracy, allowing earlier therapeutic decisions, especially in populations where genetic heterogeneity might influence disease course.

The findings from our South Asian cohort were consistent with recent global data, reinforcing the notion that anti-PLA2R antibodies serve as universally applicable predictive markers across diverse ethnic groups. However, subtle regional variations in genetic background, environmental exposure, and healthcare practices highlight the necessity for populationspecific threshold validation. Such differences may reflect demographic-specific immunological and genetic determinants, underscoring the value of tailored regional studies.

### Generalizability and Limitations

The retrospective design and single-center nature of this study are limitations that could result in selection and information bias. Because the study had a moderate sample size, especially in the non-MPR treatment groups, it had limited statistical power to perform subgroup analysis. Moreover, the follow-up was restricted to 6 months, and cutoff values suggested for anti-PLA2R antibodies should be validated externally in a larger, multicentric series.

These findings are applicable to similar South Asian populations, although their biological mechanisms are expected to be generalizable. Population-specific factors such as genetic background and healthcare access may influence external validity.

Finally, this study strengthens the argument to replace or complement traditional proteinuria-based monitoring with antibody-based paradigms. Anti-PLA2R antibodies provide direct immunological evidence of disease activity, enabling rapid and precise clinical decisions that are critical in settings where prolonged immunosuppression carries a significant risk^26^. Therefore, our findings endorse recent recommendations advocating routine anti-PLA2R measurement as a standard-of-care biomarker for the diagnosis, prognosis, and treatment guidance in PMN.

## Conclusion

This study demonstrated the clinical usefulness of quantifying anti-PLA2R antibodies in individuals with idiopathic membranous nephropathy in Pakistan. Baseline and 6-month anti-PLA2R titers were useful predictors of therapeutic response, with definite cut-off values having the ability to differentiate between remission and non-remission status. The decrease in serum albumin levels and proteinuria further augmented this predictive information, validating their combined application in maximal disease monitoring.

Our results support the integration of anti-PLA2R antibody testing into standard clinical practice, allowing personalized and accurate management of PMN. Prospective studies involving larger and more diverse populations with longer follow-up periods are recommended to validate these biomarkers for longterm prognosis and therapeutic guidance.

## Data Availability

The datasets generated and analyzed during the current study are not publicly available due to institutional policy and patient confidentiality concerns but can be accessed from the corresponding author upon reasonable request and after IRB approval.

## References

[1] Couser WG (2017). Primary membranous nephropathy. Clin J Am Soc Nephrol.

[2] Song EJ, Jeong KH, Yang YA, Lim JH, Jung HY, Choi JY (2018). Anti-phospholipase A2 receptor antibody as a prognostic marker in
patients with primary membranous nephropathy. Kidney Res Clin Pract.

[3] Cheng G, Liu J, Gilbert A, Cao Y, An C, Lv Z (2019). Serum phospholipase A2 receptor antibodies and immunoglobulin G
subtypes in adult idiopathic membranous nephropathy: clinical value
assessment. Clin Chim Acta.

[4] Timmermans SAMEG, Abdul Hamid MA, Cohen Tervaert JW, Damoiseaux JGMC, Van Paassen P, the Limburg Renal Registry (2015). Anti-PLA2R antibodies as a prognostic factor in PLA2R-related
membranous nephropathy. Am J Nephrol.

[5] Beck LH, Fervenza FC, Beck DM, Bonegio RG, Malik FA, Erickson SB (2011). Rituximab-induced depletion of anti-PLA2R autoantibodies predicts
response in membranous nephropathy. J Am Soc Nephrol.

[6] Radice A, Trezzi B, Maggiore U, Pregnolato F, Stellato T, Napodano P (2016). Clinical usefulness of autoantibodies to M-type phospholipase A2
receptor (PLA2R) for monitoring disease activity in idiopathic membranous
nephropathy (IMN). Autoimmun Rev.

[7] Pourcine F, Dahan K, Mihout F, Cachanado M, Brocheriou I, Debiec H (2017). Prognostic value of PLA2R autoimmunity detected by measurement of
anti-PLA2R antibodies combined with detection of PLA2R antigen in membranous
nephropathy: a single-centre study over 14 years. PLoS One.

[8] Seitz-Polski B, Debiec H, Rousseau A, Dahan K, Zaghrini C, Payré C (2018). Phospholipase A2 Receptor 1 epitope spreading at baseline
predicts reduced likelihood of remission of membranous
nephropathy. J Am Soc Nephrol.

[9] De Vriese AS, Glassock RJ, Nath KA, Sethi S, Fervenza FC (2017). A proposal for a serology-based approach to membranous
nephropathy. J Am Soc Nephrol.

[10] Smarz-Widelska I, Choje D, Koziol MM (2022). The Role of Anti-PLA2R and Anti-THSD7A antibodies in the
pathogenesis and diagnostics of primary membranous nephropathy: a review of
current knowledge for clinical practice. Int J Environ Res Public Health.

[11] Kukuy OL, Cohen R, Gilburd B, Zeruya E, Weinstein T, Agur T (2023). The prognostic value of anti-PLA2R antibodies levels in primary
membranous nephropathy. Int J Mol Sci.

[12] Huang Y, Zhou J, Zhou K, Huang B, Xue J, Zhang X (2022). PLA2R-IgG4 antibody as a predictive biomarker of treatment
effectiveness and prognostic evaluation in patients with idiopathic
membranous nephropathy: a retrospective study. PeerJ.

[13] Chen X, Zhang Y, Yan L, Xie Y, Li S, Zhuang Y (2024). Urine albumin-to-creatinine ratio diurnal variation rate predicts
outcomes in idiopathic membranous nephropathy. Clin Exp Nephrol.

[14] van de Logt AE, Justino J, Vink CH, van den Brand J, Debiec H, Lambeau G (2021). Anti-PLA2R1 antibodies as prognostic biomarker in membranous
nephropathy. Kidney Int Rep.

[15] van de Logt AE, Fresquet M, Wetzels JF, Brenchley P (2019). The anti-PLA2R antibody in membranous nephropathy: what we know
and what remains a decade after its discovery. Kidney Int.

[16] Hoxha E, Thiele I, Zahner G, Panzer U, Harendza S, Stahl RAK (2014). Phospholipase A2 receptor autoantibodies and clinical outcome in
patients with primary membranous nephropathy. J Am Soc Nephrol.

[17] Huang Y, Zhou J, Zhou K, Huang B, Xue J, Zhang X (2022). PLA2R-IgG4 antibody as a predictive biomarker of treatment
effectiveness and prognostic evaluation in patients with idiopathic
membranous nephropathy: a retrospective study. PeerJ.

[18] Dahan K, Debiec H, Plaisier E, Cachanado M, Rousseau A, Wakselman L, the GEMRITUX Study Group (2017). Rituximab for severe membranous nephropathy: a 6-month trial with
extended follow-up. J Am Soc Nephrol.

[19] Hanset N, Esteve E, Plaisier E, Johanet C, Michel PA, Boffa JJ (2019). Rituximab in patients with phospholipase A2 receptor– associated
membranous nephropathy and severe CKD. Kidney Int Rep.

[20] Scolari F, Delbarba E, Santoro D, Gesualdo L, Pani A, Dallera N (2021). the RI-CYCLO Investigators. Rituximab or cyclophosphamide in the
treatment of membranous nephropathy: the RI-CYCLO randomized
trial. J Am Soc Nephrol.

[21] Wang YW, Wang XH, Wang HX, Yu RH (2023). Successful treatment of patients with refractory idiopathic
membranous nephropathy with low-dose Rituximab: a single-center
experience. World J Clin Cases.

[22] Zhou K, Zhou J, Zhou L, Xue J, Liu B, Zhang Z (2024). Predictive value of the domain specific PLA2R antibodies for
clinical remission in patients with primary membranous nephropathy: a
retrospective study. PLoS One.

[23] Ronco P, Beck L, Debiec H, Fervenza FC, Hou FF, Jha V (2021). Membranous nephropathy. Nat Rev Dis Primers.

[24] Stanescu HC, Arcos-Burgos M, Medlar A, Bockenhauer D, Kottgen A, Dragomirescu L (2011). Risk HLA-DQA1 and PLA 2 R1 alleles in idiopathic membranous
nephropathy. N Engl J Med.

[25] Bullich G, Domingo-Gallego A, Vargas I, Ruiz P, Lorente-Grandoso L, Furlano M (2018). A kidney-disease gene panel allows a comprehensive genetic
diagnosis of cystic and glomerular inherited kidney diseases. Kidney Int.

[26] Bullich G, Domingo-Gallego A, Vargas I, Ruiz P, Lorente-Grandoso L, Furlano M (2020). Detection of PLA2R autoantibodies before the diagnosis of
membranous nephropathy. J Am Soc Nephrol.

