# Supplementary material for: Anti-PLA2R antibody reduction as a predictor of treatment response in primary membranous nephropathy: a retrospective study
Source: J Bras Nefrol. 2025 Dec 19;48(2):e20250228. doi: 10.1590/2175-8239-jbn-2025-0228en (PMC12716763; doi:10.1590/2175-8239-jbn-2025-0228en)
Supplement: Translation

# Translation: Redução dos níveis do anticorpo anti-PLA2R como preditor de resposta ao tratamento na nefropatia membranosa primária: um estudo retrospectivo

**DOI:** 10.1590/2175-8239-JBN-2025-0228pt

**Authors:** Khuram Bashir, Muhammad Rashid Asghar, Nayyar Saleem, Muhammad Shahzil, Aimon Riaz, Muhammad Nauman Hashmi, Ahmed Sheraz

**Affiliations:** 1 Multan Institute of Kidney Diseases, Multan, Punjab, Pakistan.; 2 Ministry of National Guard Health Affairs, Hemodialysis Care Project, Jeddah, Saudi Arabia.; 3 Punjab Employees Social Security Institution, Narowal, Punjab, Pakistan.

**Keywords:** Glomerulonefrite Membranosa, Anticorpo do Receptor da Fosfolipase A2, Doenças Glomerulares, Prognóstico, Remissão, Espontânea

## Abstract

**Introdução:**

Nefropatia membranosa primária (NMP) é uma das principais causas de síndrome nefrótica em adultos. Embora a proteinúria tenha sido oparâmetro convencional de monitoramento da doença, suas limitações como marcador inespecífico e tardio levaram ao apelo de anticorpos antireceptor de fosfolipase A2 (PLA2R) como indicadores específicos e sensíveis da atividade da doença e resposta terapêutica.

**Métodos:**

Estudo de coorte retrospectivo incluindo 60 pacientes com NMP comprovada por biópsia e positividade basal para anticorpos anti-PLA2R, tratados no *Multan Institute of Kidney Diseases*. Níveis de anti-PLA2R foram medidos no início e no acompanhamento de 6 meses, utilizando ensaio imunoenzimático (ELISA).Avaliou-se remissão clínica por critérios padrão de proteinúria e função renal. Análises estatísticas incluíram curvas ROC, regressão logística, análises de correlação.

**Resultados:**

Níveis basais de anti-PLA2R foram significativamente menores em pacientes que alcançaram remissão (p = 0,047), e níveis aos 6 meses apresentaram forte correlação com a resposta terapêutica (p < 0,001). Análise ROC demonstrou boa precisão preditiva (AUC de 0,707 [basal] e 0,815 [6 meses]), identificando pontos de corte ideais para remissão em 125,5 RU/mL e 37,75 RU/mL, respectivamente. A análisede regressão logística confirmou nível basal de albumina, anti-PLA2R aos 6 meses e redução da proteinúria como preditores independentes de remissão.

**Conclusão:**

Níveis de anticorpos anti-PLA2R no período basal e aos 6 meses são preditores robustos de resposta terapêutica na NMP, superando a proteinúria na avaliação precoce da remissão. Esses achados sustentam a integração do monitoramento do anti-PLA2R à prática clínica padrão para o manejo personalizado e ajuste terapêutico na NMP.

## Introdução

A nefropatia membranosa primária (NMP) é uma das principais causas de síndrome nefrótica em adultos e caracteriza-se pela deposição de imunocomplexos na membrana basal glomerular, resultando em proteinúria e potencial progressão para doença renal em estágio terminal (DRET)^1^. Marcadores prognósticos precisos são cruciais para orientar o manejo clínico. No entanto, a dependência exclusiva da proteinúria apresenta limitações significativas. Embora a proteinúria seja uma característica marcante da NMP, trata-se de um marcador inespecífico, influenciado por fatores extrínsecos, como ingestão alimentar de proteínas, variações hemodinâmicas e doenças concomitantes, o que reduz sua confiabilidade para o monitoramento da doença^2^. A necessidade de biomarcadores mais precisos tem levado a um interesse crescente pelos anticorpos anti-receptor da fosfolipase A2 (PLA2R), que avançaram de forma significativa a avaliação diagnóstica e prognóstica da NMP.

A identificação do PLA2R como um importante antígeno-alvo na NMP aprimorou o panorama da nefrologia diagnóstica. Os anticorpos anti-PLA2R são detectados em aproximadamente 85% dos casos de NMP, tornando-os biomarcadores altamente específicos para a doença^3^. Ao contrário da proteinúria, que reflete danos glomerulares a jusante, os anticorpos anti-PLA2R estão diretamente envolvidos na patogênese da NMP, oferecendo uma representação mais precisa da atividade da doença. Seus níveis se correlacionam com o curso clínico, incluindo soropositividade durante a fase ativa da doença, declínio dos títulos na remissão e reaparecimento durante a recidiva^4^. Essa relação dinâmica torna os anticorpos anti-PLA2R biomarcadores ideais para o monitoramento da doença e a estratificação de risco.

A proteinúria pode permanecer elevada mesmo após o tratamento bem-sucedido, levando a uma imunossupressão prolongada. Isso ocorre porque ela pode não estar correlacionada com precisão à atividade imunológica subjacente que causa a doença. Por exemplo, estudos demonstraram que a proteinúria pode persistir apesar da resolução do depósito de imunocomplexos^5^. Por outro lado, os anticorpos anti-PLA2R fornecem um reflexo mais preciso da atividade da doença, com estudos demonstrando que uma redução de 50% nos níveis de anticorpos dentro de dois meses após o início do tratamento está associada a desfechos favoráveis^6^. Além disso, níveis basais mais elevados de anticorpos anti-PLA2R têm sido associados a um prognóstico mais desfavorável e a uma progressão mais rápida para DRET, destacando sua utilidade na estratificação de risco e no planejamento terapêutico personalizado^7,8^.

A NMP é tratada com terapias imunossupressoras; entretanto, a falta de biomarcadores confiáveis para monitorar a eficácia do tratamento dificulta a tomada de decisões clínicas. A medição dos anticorpos anti-PLA2R oferece uma solução promissora, uma vez que fornece informações precoces sobre a resposta ao tratamento. Por exemplo, uma redução de 90% nos níveis de anti-PLA2R pode justificar a suspensão da terapia imunossupressora, minimizando, assim, os riscos associados ao uso prolongado desses medicamentos^9^.

Apesar desses avanços, ainda existe uma lacuna crítica em nossa compreensão da correlação entre os níveis de anticorpos anti-PLA2R e os desfechos do tratamento, especialmente em populações diversas. A maioria dos estudos foi realizada em coortes ocidentais, deixando amplamente inexplorada a influência de fatores genéticos, ambientais e de estilo de vida em outras regiões. Meta-análises corroboram o valor diagnóstico e prognóstico dos anticorpos anti-PLA2R, demonstrando sua elevada especificidade e sensibilidade na diferenciação entre casos de NMP relacionados e não relacionados ao PLA2R, bem como na predição de remissão espontânea^10,11,12^.

Este estudo teve como objetivo abordar essas lacunas ao investigar a relação entre níveis de anticorpos anti-PLA2R e desfechos clínicos em pacientes com NMP na região de Multan, no Paquistão. Nossa hipótese foi a de que os títulos de anticorpos anti-PLA2R se correlacionam com a atividade da doença, a resposta ao tratamento e os desfechos renais em longo prazo, fornecendo assim uma ferramenta confiável para o monitoramento personalizado da doença. Além disso, nosso objetivo foi comparar a eficácia do tratamento entre diferentes regimes imunossupressores quanto aos níveis de anticorpos, gerando possibilidades específicas por região sobre o manejo da NMP. Ao preencher essas lacunas, este estudo buscou refinar as estruturas prognósticas e terapêuticas para a NMP em populações diversas. Este trabalho adotou um desenho de coorte retrospectivo, em conformidade com as diretrizes STROBE.

## Métodos

### Critérios de Inclusão e Exclusão

Este estudo de coorte retrospectivo foi realizado no *Multan Institute of Kidney Diseases* (MIKD). Os critérios de inclusão foram: diagnóstico com base em biópsia renal e achados de imunocoloração consistentes com NMP; positividade sérica para anticorpos anti-PLA2R no período basal; diagnóstico e tratamento entre agosto de 2017 e junho de 2023 no MIKD; pacientes em terapia imunossupressora, particularmente aqueles classificados como de risco moderado ou alto para progressão da doença (ou seja, com pouca probabilidade de remissão espontânea); disponibilidade de títulos de anticorpos anti-PLA2R no período basal e no acompanhamento de 6 meses; e disposição para participar e cumprir com os requisitos do protocolo do estudo.

Os critérios de exclusão para a pesquisa foram: positividade para infecções conhecidas por causarem NM secundária (hepatite B ou C positivas); qualquer paciente com malignidade ativa diagnosticada durante a avaliação ou o acompanhamento; exposição a toxinas ou medicamentos reconhecidamente associados à NM secundária (por exemplo, AINEs, sais de ouro e penicilamina); ausência dos níveis de anticorpos anti-PLA2R tanto no período basal quanto no acompanhamento de 6 meses; e pacientes que não quisessem ou não pudessem respeitar o acompanhamento ou o protocolo do estudo.

O viés de seleção foi minimizado ao incluir todos os pacientes elegíveis que foram consecutivamente diagnosticados com NMP comprovada por biópsia e positividade para PLA2R entre agosto de 2017 e junho de 2023. O viés de medição foi reduzido por meio do uso de protocolos padrão de ensaio imunoenzimático (ELISA) e pela atuação de profissionais de laboratório cegados para os desfechos clínicos.

### Pacientes e amostras de sangue

Dos 112 pacientes avaliados quanto à elegibilidade, 52 foram excluídos devido a resultados negativos ou ausentes para anticorpos anti-PLA2R, causas secundárias de nefropatia membranosa, acompanhamento incompleto ou perda de acompanhamento. Um total de 60 pacientes preencheram os critérios de inclusão e foram analisados. Estes foram posteriormente estratificados por regime terapêutico nos grupos *Ponticelli* Modificado (n = 46), Rituximabe (n = 9) e *Cattran* (n = 5). Todo o processo de seleção de pacientes e estratificação do tratamento está descrito na Figura 1. Este estudo foi analisado e aprovado como sendo de “não risco“ ou “de baixo risco” (*IRB-exempt*) pelo Comitê de Ética em Pesquisa do *Indus Hospital and Health Network* (IHHN), Paquistão, sob a categoria de isenção 4, para pesquisas secundárias envolvendo dados clínicos e amostras existentes, sem contato direto com os participantes. A pesquisa seguiu os princípios éticos descritos na Declaração de *Helsinque* e no Relatório *Belmont*, e todos os dados foram tratados em conformidade com os padrões estabelecidos de conduta responsável em pesquisa.

**Figura 1.** Dos 112 pacientes avaliados para elegibilidade, 52 foram excluídos devido à negatividade ou à ausência de resultados para anticorpos anti-PLA2R, causas secundárias de nefropatia membranosa, dados incompletos ou perda de acompanhamento. A coorte final incluiu 60 pacientes, todos preenchendo os critérios de inclusão e sendo analisados. Os pacientes foram estratificados em três grupos de tratamento: *Ponticelli* Modificado (n = 46), Rituximabe (n = 9) e *Cattran* (n = 5).

### Regimes Terapêuticos

Os regimes terapêuticos foram definidos em colaboração com nefrologistas consultores, após considerar as preferências dos pacientes. Foi obtido o consentimento informado por escrito de todos os participantes. O regime *Ponticelli* Modificado (MPR) foi administrado a 46 pacientes, enquanto nove receberam rituximabe e cinco foram submetidos ao regime de *Cattran*.

### Critérios de Remissão

A remissão completa (RC) foi considerada quando o paciente apresentou proteinúria urinária de 24 horas ≤0,3 g/dia, com função renal estável ou melhorada em relação ao valor basal nas avaliações de acompanhamento. A remissão parcial (RP) foi considerada quando houve proteinúria urinária de 24 horas <3,5 g/dia, com redução de pelo menos 50% juntamente com função renal estável. A ausência de resposta foi considerada quando houve proteinúria urinária de 24 horas >3,5 g/dia ou piora da creatinina sérica^13^.

### Detecção Quantitativa de Anticorpos PLA2R

As amostras séricas foram analisadas por meio de ELISA quantitativo.

### Análise Estatística

A análise estatística incluiu estatísticas descritivas para resumir as variáveis basais e de acompanhamento. Testes qui-quadrado foram utilizados para avaliar as relações entre variáveis binárias, enquanto testes t independentes foram empregados para verificar as diferenças médias entre os grupos de remissão e não remissão para variáveis contínuas. A análise da curva ROC (*Receiver Operating Characteristic*) avaliou o desempenho preditivo dos níveis de anti-PLA2R, e o índice de *Youden* foi aplicado para determinar os valores de corte ideais. A regressão logística foi utilizada para identificar fatores de risco independentes para o estado de remissão, enquanto o coeficiente de correlação de *Pearson* foi empregado para avaliar a magnitude e o padrão das relações entre as variáveis clínicas e os desfechos de remissão. Para a análise categórica, os títulos basais de anti-PLA2R foram classificados como baixos (<125,5 RU/mL) ou elevados (≥125,5 RU/mL). O tamanho da amostra foi modesto, especialmente no grupo de tratamento não submetido ao regime MPR, o que limitou o poder estatístico da análise de subgrupos.

## Resultados

### Características dos Pacientes

As características basais dos 60 pacientes incluídos neste estudo estão resumidas nas Tabelas 1 e 2. A coorte foi composta por 75,0% de indivíduos do sexo masculino (n = 45). A maioria dos pacientes foi classificada como portadora de DRC estágio 1 (68,3%; n = 41).

**Tabela 1:** Descrição das variáveis categóricas

Variável	Categoria	Frequência (n)	Porcentagem (%)
Sexo	Masculino	45	75,0
	Feminino	15	25,0
Estágio da DRC	Estágio 1	41	68,3
	Estágio 2	13	21,7
	Estágio 3	5	8,3
	Estágio 4	1	1,7
FIAT	N/A	24	40,0
	Leve	31	51,7
	Moderada	5	8,3
Regime Terapêutico	Regime de *Ponticelli* Modificado (MPR)	46	76,7
	Rituximabe	9	15,0
	*Cattran*	5	8,3
Hipertensão	Não	45	75,0
	Sim	15	25,0
Categorias Basais de Anti-PLA2R	Baixa Positividade	31	51,7
	Elevada Positividade	29	48,3

**Tabela 2:** Descrição das variáveis contínuas

Parâmetro	Média	Desvio padrão
Idade (anos)	33,13	12,15
TFGe Basal (mL/min/1,73m^2^)	107,32	41,71
Anti-PLA2R Basal (RU/mL)	200,84	182,02
Proteinúria Basal (g/dia)	8,17	4,07
Albumina Basal (g/dL)	2,17	0,66
Creatinina Basal (mg/dL)	0,96	0,64
TFGe aos 6 meses (mL/min/1,73m^2^)	105,12	39,38
Creatinina aos 6 meses (mg/dL)	1,02	0,86
Anti-PLA2R aos 6 meses (RU/mL)	48,87	80,64
Proteinúria aos 6 meses (g/dia)	4,43	3,89
Albumina aos 6 meses (g/dL)	3,37	0,90

A fibrose intersticial e atrofia tubular (FIAT) foi leve em 51,7% (n = 31) dos pacientes e moderada em 8,3% (n = 5). A hipertensão esteve presente em 25,0% (n = 15) dos pacientes. Não foram observados dados ausentes para as variáveis primárias utilizadas nesta análise.

### Análise Entre Diferentes Grupos de Tratamento

Quanto ao tratamento, 76,7% (n = 46) dos pacientes receberam MPR, 15,0% (n = 9) receberam rituximabe e 8,3% (n = 5) foram tratados com o regime de *Cattran*. Os níveis basais de anticorpos anti-PLA2R foram classificados como positividade baixa em 51,7% (n = 31) e positividade elevada em 48,3% (n = 29) dos pacientes.

A média da TFGe basal (EPI-CKD) foi de 107,32 ± 41,71 mL/min, enquanto a média da proteinúria basal foi de 8,17 ± 4,07 g/dia. O nível médio de albumina sérica no período basal foi de 2,17 ± 0,66 g/dL, e o nível médio basal de creatinina foi de 0,96 ± 0,64 mg/dL. O nível médio basal de anti-PLA2R foi de 200,84 ± 182,02 RU/mL.

#### Biomarcadores do acompanhamento de seis meses

No acompanhamento de seis meses, a TFGe média foi de 105,12 ± 39,38 mL/min, e o nível médio de proteinúria diminuiu para 4,43 ± 3,89 g/dia. A média da albumina sérica aumentou para 3,37 ± 0,90 g/dL, enquanto a média do nível de anti-PLA2R baixou para 48,87 ± 80,64 RU/mL.

### Fatores Para Remissão

#### Análise bivariada

Testes qui-quadrado foram utilizados para avaliar variáveis categóricas. Não foram encontradas associações significativas entre remissão e tipo de tratamento (χ^2^ = 1,85; p = 0,397), sexo (χ^2^ = 1,09; p = 0,296), FIAT (χ^2^ = 1,09; p = 0,579), ou hipertensão (χ^2^ = 0,02; p = 0,881). No entanto, as categorias basais de anti-PLAR apresentaram uma associação estatisticamente significativa com a remissão (χ^2^ = 9,67; p = 0,002).

#### Comparação de variáveis contínuas

Testes t independentes foram utilizados para comparar variáveis contínuas entre os grupos remissão e não remissão. Os níveis basais de anti-PLA2R foram significativamente mais baixos no grupo remissão (t = ”2,03; p = 0,047), enquanto os níveis basais de albumina foram significativamente mais elevados (t = 2,24; p = 0,029). No entanto, idade (t = 1,15; p = 0,253), estágio da DRC (t = ”0,92; p = 0,363), TFGe basal (t = 0,18; p = 0,86), proteinúria basal (t = ”0,37; p = 0,714) e nível basal de creatinina (t = ”0,24, p = 0,811) não apresentaram diferenças significativas entre os grupos (Tabela 3).

**Tabela 3:** Teste de significância e análise de correlação das variáveis clínicas com a remissão na nefropatia membranosa primária

Variável	Teste estatístico	Valor de p	Coeficiente de correlação	Valor de p
Tratamento	1,85	0,397	–0,081	0,539
Sexo	1,09	0,296	0,135	0,305
FIAT	1,09	0,579	0,079	0,550
Hipertensão	0,02	0,881	–0,019	0,884
Idade em anos	1,15	0,253	0,138	0,294
Estágio da DRC	–0,92	0,363	–0,098	0,458
TFGe (EPI-CKD)	0,18	0,860	–0,013	0,924
Anti-PLA2R Basal (RU/mL)	–2,03	0,047	–0,359	0,005
Proteinúria Basal (g/dia)	–0,37	0,714	–0,024	0,855
Albumina Basal (g/dL)	2,24	0,029	0,265	0,040
Creatinina Basal (mg/dL)	–0,24	0,811	–0,070	0,597
TFG aos 6 meses (mL/min)	0,82	0,418	0,101	0,442
Creatinina aos 6 meses (mg/dL)	–1,36	0,180	–0,186	0,155
Anti-PLA2R aos 6 meses (RU/mL)	–3,67	0,001	–0,547	<0,001
Proteinúria aos 6 meses (g/dia)	–8,45	<0,001	–0,866	<0,001
Albumina aos 6 meses (g/dL)	6,56	<0,001	0,663	<0,001

### Análise do Acompanhamento de Seis Meses

Aos seis meses, foram observadas diferenças significativas nos níveis de anti-PLA2R no acompanhamento (t = –3,67; p = 0,001), na proteinúria (t = –8,45; p < 0,001) e nos níveis de albumina (t = 6,56; p < 0,001), todos fortemente associados à remissão. As alterações na proteinúria entre o período basal e os seis meses também foram significativamente associadas à remissão (t = 4,37; p <001). No entanto, as alterações no anti-PLA2R (t = ”0,58; p = 0,564) e na TFG (t = –0,8; p = 0,429) não foram estatisticamente significativas.

### Análise de Correlação

Os coeficientes de correlação de Pearson foram calculados para avaliar a relação entre remissão e variáveis clínicas. Os níveis basais de anti-PLA2R apresentaram correlação negativa com a remissão (r = –0,359; p = 0,005), enquanto os níveis basais de albumina apresentaram correlação positiva (r = 0,265; p = 0,040). Aos seis meses, foram observadas fortes correlações entre a remissão e os níveis de anti-PLA2R no acompanhamento (r = –0,547; p < 0,001), proteinúria (r = –0,866; p < 0,001) e albumina (r = 0,663;p < 0,001). Além disso, as alterações na proteinúria entre o período basal e os seis meses demonstraram uma correlação positiva significativa com a remissão (r = 0,484; p < 0,001).

Outras variáveis, incluindo idade, sexo, estágio da DRC, TFGe, FIAT, hipertensão, complicações e tratamento, não apresentaramcorrelações estatisticamente significativas com a remissão (p > 0,05). Os achados de significância e correlação são apresentados de forma detalhada na Tabela 3.

### Análise da Curva ROC

#### Níveis basais de anticorpos anti-PLA2R

A curva ROC foi utilizada para avaliar os níveis basais de anticorpos anti-PLA2R na predição da remissão em pacientes com nefropatia membranosa idiopática (NMI). A área sob a curva (AUC) foi de 0,707 (IC 95%: 0,570–0,845), indicando poder discriminatório moderado e capacidade preditiva significativa (p = 0,006), conforme demonstrado na Figura 2. Utilizando o índice de *Youden*, o ponto de corte ideal para predição de remissão foi de 125,5 RU/mL, o que resultou em sensibilidade e especificidade de 69,0% e 71,0%, respectivamente.

**Figura 2.** Curva ROC demonstrando níveis basais de Anti-PLA2R como preditor de remissão na nefropatia membranosa idiopática. A AUC foi de 0,707 (IC 95%: 0,570–0,845; p = 0,006), indicando precisão diagnóstica moderada. Um ponto de corte ideal de 125,5 RU/mL apresentou sensibilidade de 69,0% e especificidade de 71,0%.

### Níveis de Anticorpos Anti-PLA2R no Acompanhamento de 6 Meses

A análise ROC para os níveis de anti-PLA2R aos 6 meses apresentou uma AUC de 0,815 (IC 95%: 0,706–0,923), indicando sólida precisão diagnóstica (p < 0,001), conforme demonstrado na Figura 3. Utilizando o índice de *Youden*, o ponto de corte ideal foi determinado em 37,75 RU/mL,o que proporcionou sensibilidade de 93,1% e especificidade de 54,8%.

**Figura 3.** Curva ROC avaliando níveis de anti-PLA2R aos 6 meses como preditor de remissão na nefropatia membranosa idiopática. AUC = 0,815 (IC 95%: 0,706–0,923; p < 0,001), indicando elevada precisão diagnóstica. O ponto de corte ideal de 37,75 RU/mL apresentou sensibilidade de 93,1% e especificidade de 54,8%.

### Análise de Regressão

A regressão logística multivariada foi utilizada para identificar os principais preditores de remissão em pacientes com NMI. Um nível basal mais elevado de albumina sérica foi associado a um aumento de 167% nas chances de remissão por g/dL (p = 0,041; Exp(B) = 2,669), o qual aumentou 12 vezes em seis meses (p < 0,001). Níveis mais baixos de anti-PLA2R aos seis meses também aumentaram as chances de remissão, com cada redução de 1 RU/mL elevando as probabilidades em 2,7% (p = 0,009; Exp(B) = 0,973). Por outro lado, níveis basais mais elevados de Log anti-PLA2R reduziram as chances de remissão em 83,5% (p = 0,012; Exp(B) = 0,165), indicando seu valor como biomarcador. Além disso, cada redução de 1 g/dia na proteinúria aumentou as chances de remissão em 32% (p = 0,001; Exp(B) = 1,319). Os resultados são apresentados na Tabela 4.

**Tabela 4:** Resultados da análise de regressão das variáveis significativas

Variável Preditiva	Valor de p	Exp(B)
Albumina Basal (g/dL)	0,041	2,669
Anti-PLA2R aos 6 meses (RU/mL)	0,009	0,973
Anti-PLA2R Basal	0,012	0,165
Albumina aos 6 meses (g/dL)	<0,001	11,990
Alteração na Proteinúria (g/dia)	0,001	1,319

## Discussão

Este estudo ressalta o papel fundamental dos níveis de anticorpos anti-PLA2R na predição da resposta ao tratamento e dos desfechos de longo prazo em pacientes com NMP em Multan, Paquistão. Nossos achados corroboram evidências globais que destacam a utilidade dos anticorpos anti-PLA2R como biomarcadores para a atividade e remissão da doença, ao mesmo tempo em que oferecem possibilidades específicas da região sobre a eficácia do tratamento e a estratificação prognóstica^11,14,15^.

### Anticorpos anti-PLA2R como Biomarcador Prognóstico

Constatamos que os níveis basais de anticorpos anti-PLA2R foram significativamente mais baixos em pacientes que alcançaram remissão (p = 0,047), corroborando estudos anteriores que indicavam que títulos basais mais elevados estavam associados a piores desfechos^5,16^. A análise da curva ROC confirmou o poder preditivo dos níveis de anti-PLA2R, com uma AUC basal de 0,707 (p = 0,006) e um ponto de corte ideal de 125,5 RU/mL (sensibilidade de 69,0% e especificidade de 71,0%). Notavelmente, os níveis do acompanhamento de 6 meses apresentaram um valor discriminatório ainda mais robusto (AUC = 0,815; p < 0,001), com ponto de corte de 37,75 RU/mL (sensibilidade, 93,1%; especificidade, 54,8%). Esses resultados são consistentes com os de Huang et al.^17^, que relataram uma AUC de 0,970 para PLA2R-IgG4 na predição de remissão, reforçando, assim, a relevância do monitoramento de anticorpos em populações diversas.

### Correlação com Parâmetros Clínicos

As reduções médias significativas nos níveis de anti-PLA2R (-152,02 ± 165,91 RU/mL) e na proteinúria (-3,74 ± 5,40 g/dia) ao longo de seis meses refletem a estreita relação entre os títulos de anticorpos e a atividade da doença. A regressão logística identificou os níveis basais de albumina sérica (*odds ratio* (OR) = 2,669; p = 0,041) e os níveis de anti-PLA2R aos seis meses (OR = 0,973; p = 0,009) como preditores independentes de remissão. Esses resultados ecoam os de Dahan et al.^18^, que constataram que a remissão sorológica frequentemente precede a resposta clínica, sustentando o ajuste precoce do tratamento com base nos níveis de anticorpos. Curiosamente, a alteração isolada nos níveis de anti-PLA2R não foi um preditor significativo, sugerindo que os limites absolutos eram clinicamente mais confiáveis do que as alterações relativas.

### Eficácia do Tratamento Entre os Regimes

Em nossa coorte, 76,7% dos pacientes receberam o regime *Ponticelli* Modificado (MPR), 15,0% foram tratados com rituximabe e 8,3% receberam o regime de *Cattran*. Embora não tenha sido observada diferença estatisticamente significativa nas taxas de remissão entre os grupos de tratamento (p = 0,397), a redução consistente nos níveis de anti-PLA2R e na proteinúria em todos os grupos indicou uma ampla eficácia imunossupressora. Esses achados estão em consonância com os de Huang et al.^17^, que relataram taxas de remissão semelhantes às do rituximabe e da ciclofosfamida (74% vs. 67,5%; p = 0,114). De modo semelhante, o ensaio clínico randomizado RI-CYCLO demonstrou que ambas as terapias alcançaram taxas de remissão comparáveis em 24 meses, com a ciclofosfamida apresentando uma taxa mais elevada de remissão completa aos 12 meses^19,20,21,22^. No entanto, o pequeno tamanho amostral de regimes não MPR limita conclusões definitivas e ressalta a necessidade de estudos comparativos mais amplos em populações do sul da Ásia.

### Análise da Curva ROC e Utilidade Clínica

A análise ROC resultou em valores preditivos moderados a elevados para os níveis de anti-PLA2R, com AUCs de 0,707 (basal) e 0,815 (6 meses). Isso significa que, no período basal, houve uma chance 70,7% maior e, aos seis meses, uma probabilidade 81,5% mais elevada de que os pacientes que alcançaram remissão apresentassem níveis mais baixos de anti-PLA2R em comparação àqueles sem remissão, ressaltando o valor preditivo desse biomarcador. Os valores de corte de 125,5 RU/mL no período basal e de 37,75 RU/mL no acompanhamento ofereceram um equilíbrio ideal entre sensibilidade e especificidade. Esses resultados refletem os achados de coortes ocidentais e confirmam que a dinâmica do anti-PLA2R mantém sua relevância clínica em diferentes populações. Nosso estudo preenche uma lacuna crítica na literatura ao contribuir com dados provenientes de um contexto sul-asiático, onde tais evidências são escassas.

Além disso, este trabalho corrobora o consenso crescente de que a proteinúria, embora há muito utilizada como ferramenta de monitoramento, apresenta limitações em razão de sua resposta tardia e natureza inespecífica^19^. Em contrapartida, os anticorpos anti-PLA2R fornecem informações em tempo real sobre a atividade da doença, permitindo decisões terapêuticas mais precoces e precisas, incluindo intensificação, redução e descontinuação da imunossupressão.

### Avanços Recentes e Análises Comparativas

Os avanços na imunopatologia aprofundaram substancialmente nossa compreensão da NMP, direcionando a prática clínica para paradigmas da medicina de precisão orientados por biomarcadores, como os anticorpos anti-PLA2R^23^. Estudos recentes têm enfatizado a integração de marcadores sorológicos com predisposições genéticas, particularmente polimorfismos genéticos do PLA2R1, sugerindo que a combinação de perfis sorológicos e genéticos pode aprimorar de forma significativa a estratificação de risco^24,25^. Os polimorfismos no gene PLA2R1 têm sido associados a variações na produção de anticorpos e na gravidade da doença, o que implica que a composição genética dos pacientes pode influenciar os desfechos terapêuticos independentemente dos títulos sorológicos^8^. A incorporação dessas informações genéticas em algoritmos clínicos pode aumentar a precisão preditiva, possibilitando decisões terapêuticas mais precoces, especialmente em populações onde a heterogeneidade genética pode influenciar o curso da doença.

Os achados da nossa coorte do sul da Ásia foram consistentes com dados globais recentes, reforçando a noção de que os anticorpos anti-PLA2R atuam como marcadores preditivos universalmente aplicáveis em diferentes grupos étnicos. No entanto, variações regionais sutis no antecedente genético, na exposição ambiental e nas práticas de saúde ressaltam a necessidade de validação de limites específicos para cada população. Tais diferenças podem refletir determinantes imunológicos e genéticos específicos de cada grupo demográfico, destacando o valor de estudos regionais personalizados.

### Generalização e Limitações

O desenho retrospectivo e o caráter de centro único deste estudo constituem limitações que podem resultar em vieses de seleção e de informação. Como o estudo teve um tamanho amostral moderado, especialmente nos grupos de tratamento que não utilizaram o MPR, ele apresentou poder estatístico limitado para realizar análises de subgrupos. Além disso, o acompanhamento foi restrito a 6 meses, e os valores de corte sugeridos para anticorpos anti-PLA2R devem ser validados externamente em uma série multicêntrica e mais ampla.

Esses achados são aplicáveis a populações semelhantes do sul da Ásia, embora se espere que seus mecanismos biológicos sejam generalizáveis. Fatores específicos à população, como antecedentes genéticos e acesso a serviços de saúde, podem influenciar a validade externa.

Por fim, este estudo reforça o argumento em favor da substituiçãoou complementaçãodomonitoramento tradicional baseado na proteinúria por paradigmas fundamentados em anticorpos. Os anticorpos anti-PLA2R fornecem evidência imunológica direta da atividade da doença, permitindo decisões clínicas rápidas e precisas, essenciais em contextos nos quais a imunossupressão prolongada acarreta riscos significativos^26^. Portanto, nossos achados endossam recomendações recentes que defendem a medição de rotina do anti-PLA2R como um biomarcador padrão de cuidado para o diagnóstico, prognóstico e orientação do tratamento na NMP.

## Conclusão

Este estudo demonstrou a utilidade clínica da quantificação dos anticorpos anti-PLA2R em indivíduos com nefropatia membranosa idiopática no Paquistão. Os títulos de anti-PLA2R no período basal e aos 6 meses foram preditores úteis da resposta terapêutica, com valores de corte definidos capazes de diferenciar entre os estados de remissão e não remissão. A redução nos níveis séricos de albumina e na proteinúria aumentou ainda mais essa informação preditiva, validando sua aplicação combinada no monitoramento otimizado da doença.

Nossos resultados apoiam a integração do teste de anticorpos anti-PLA2R à prática clínica padrão, possibilitando o manejo personalizado e preciso da NMP. Recomenda-se a realização de estudos prospectivos envolvendo populações maiores e mais diversificadas, com períodos de acompanhamento mais longos, visando validar esses biomarcadores para o prognóstico de longo prazo e orientação terapêutica.

## Data Availability

Os conjuntos de dados gerados e analisados durante o presente estudo não estão disponíveis publicamente devido a políticas institucionais e questões de confidencialidade dos pacientes, mas podem ser acessados junto ao autor correspondente mediante solicitação razoável e após aprovação do Comitê de Ética em Pesquisa (IRB).
